# Removal of 16 pesticide residues from strawberries by washing with tap and ozone water, ultrasonic cleaning and boiling

**DOI:** 10.1007/s10661-015-4850-6

**Published:** 2015-12-22

**Authors:** Bozena Lozowicka, Magdalena Jankowska, Izabela Hrynko, Piotr Kaczynski

**Affiliations:** Plant Protection Institute - National Research Institute, Chelmonskiego 22, 15-195 Bialystok, Poland

**Keywords:** Processing, Fungicide and insecticide residues, Strawberries, Risk assessment, PCA

## Abstract

The effects of washing with tap and ozone water, ultrasonic cleaning and boiling on 16 pesticide (ten fungicides and six insecticides) residue levels in raw strawberries were investigated at different processing times (1, 2 and 5 min). An analysis of these pesticides was conducted using gas chromatography with nitrogen-phosphorous and electron capture detection (GC-NPD/ECD). The processing factor (PF) for each pesticide in each processing technique was determined. Washing with ozonated water was demonstrated to be more effective (reduction from 36.1 to 75.1 %) than washing with tap water (reduction from 19.8 to 68.1 %). Boiling decreased the residues of the most compounds, with reductions ranging from 42.8 to 92.9 %. Ultrasonic cleaning lowered residues for all analysed pesticides with removal of up to 91.2 %. The data indicated that ultrasonic cleaning and boiling were the most effective treatments for the reduction of 16 pesticide residues in raw strawberries, resulting in a lower health risk exposure. Calculated PFs for alpha-cypermethrin were used to perform an acute risk assessment of dietary exposure. To investigate the relationship between the levels of 16 pesticides in strawberry samples and their physicochemical properties, a principal component analysis (PCA) was performed.

**Graphical abstract:**
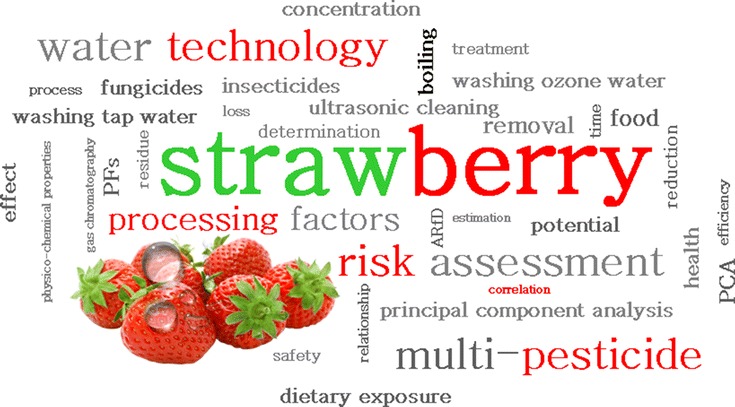
ᅟ

ᅟ

## Introduction

The health benefits of strawberries are well-known. Strawberries (*Fragaria ananassa*) play an important role in human nutrition, and they are a valuable fruit in our diet. Strawberries are low in calories (32 cal/100 g) and fats but are a rich source of health-promoting phytonutrients, minerals, and vitamins that are essential for optimum health. Additionally, strawberries have relatively high amounts of phenolic flavonoid phytochemicals called anthocyanins and ellagic acid. Scientific studies show (Battino et al. 2009; McDougall and Stewart 2012) that consumption of these berries may have potential health benefits against cancer, aging, inflammation and neurological diseases. Strawberry has an ORAC value (oxygen radical absorbance capacity, a measure of anti-oxidant strength) of approximately 3577 μmol Trolox equivalent (TE) per 100 g. Moreover, the fresh berries are an excellent source of vitamin C (100 g provides 58.8 mg or approximately 98 % of recommended dietary allowance (RDI)), as well as A, E and B-complex vitamins (the latter are also a powerful natural antioxidants).

However, during their very short fruiting time, strawberries are susceptible to several fungal diseases (*Botrytis cinerea* Pers, *Mycosphaerella fragariae* and *Erysiphales*, *etc*.) and insect pests (*Otiorhynchus* spp., *Anthonomus rubi*, *etc*.). Therefore, fungicides and insecticides are widely used. Pesticides have different modes of action and may move in various ways after they come in contact with the plant. Pesticides with systemic action are absorbed through the leaves, stems or roots and then transported within the treated plant by the plant’s vascular system. Contact pesticides are applied to surfaces of plants and must come into direct contact with the pest to be effective (IPM 2012).

Several studies have reported the presence of various types of pesticide residues in strawberries (Fernandes et al. 2012; Wołejko et al. 2014), sometimes above permitted MRLs maximum residue levels (MRLs), and their impacts on human health. However, there are very few data regarding methods of eliminating multi-class pesticides from strawberries (Kim and Huat 2010; Angioni et al. 2004; Christensen et al. 2003).

Certain types of processing may reduce pesticide residues. To reduce concentrations or remove pesticide residues from agricultural products, some methods and supporting equipment have been developed, such as washing, refrigeration, peeling, cooking, ozone treatment and ultrasonic waves (Shabeer et al. 2015; Kentish and Feng 2014; Kaushik et al. 2009; Keikotlhaile et al. 2010). Ozone is a natural substance in the atmosphere and is one of the most potent sanitizers against a wide spectrum of microorganisms (Khadre et al. 2001), whereas ultrasonic waves cause a phenomenon known as cavitation, which is the rapid formation and violent collapse of micron-sized bubbles in a liquid medium, causing tiny implosions that provide the cleaning power (Kentish and Feng 2014). Generally, the degradation of dissolved organic compounds in aqueous media involves pyrolysis inside the bubble and/or within the bubble-bulk interface region, and free-radical-mediated reactions occur in the bubble-bulk interface region and/or in the bulk liquid (Adewuyi 2001).

However, the effect of processing on the reduction of pesticides in berry fruits has rarely been studied. There has been some research on the removal of pesticide residues from apples (Han et al. 2013; Rawn et al. 2008; Ong et al. 1996), carrots (Bonnechère et al. 2012; Burchat et al. 1998), grapes (Shabeer et al. 2015; Cabras et al. 2000), melons (Bonnechère, Hanot, Jolie et al. 2012a; Krol et al. 2000) and spinach (Bonnechère, Hanot, Jolie et al. 2012b) by home and industrial processing, but the literature on strawberries is limited.

Over the last few years, pesticide residues resulting from the use of synthetic plant protection products in fruit farming are of major concern to consumers, particularly for children (Jurewicz and Hanke 2008), due to their harmful effects (Keikotlhaile and Spanoghe 2011). Consuming raw fruit or the corresponding processed commodities with pesticide residues above permitted tolerances (maximum residue levels, MRLs) can be a significant route to human health exposure (Łozowicka et al. 2013; Aktar et al. 2009). Knowledge of the effects of food processing on the level of pesticide residues in fruits is required to reduce dietary exposure (Keikotlhaile et al. 2010).

The behaviour of pesticide residue is related to the physicochemical properties, not only processing methods but also to the time of application, the meteorological condition during the cultivation, and the commodity (Holland et al. 1994). Processing factor (PF) is a main factor describing processing efficiency. Many PFs remain unknown; therefore, this value for particular combination pesticide/processing techniques/matrix need to be determined. It becomes important when researchers want to perform a risk assessment for a pesticide under a specific treatment in specific commodity. MRLs are often only available for raw commodities (e.g., apples, grapes) and not for the corresponding processed products (e.g., juice, purée, pomace, raisins). If the residue level in a processed product is needed for a risk assessment, the residue in the raw product could be simply multiplied by the appropriate PF.

In our study, washing with tap and ozone water, supported by ultrasonic waves and boiling, was applied to evaluate the effects on the removal of 16 pesticide residues including ten fungicides (boscalid, bupirimate, cyprodinil, fenhexamid, fludioxonil, folpet, iprodione, pyraclostrobin, tetraconazole and trifloxystrobin) and six insecticides (acetamiprid, alpha-cypermethrin, chlorpyrifos, deltamethrin, lambda-cyhalothrin and pirimicarb) in raw strawberries. The model compounds were chosen based on results from previous studies (Łozowicka et al. 2012a, b, 2013), and the results of an analysis conducted every year in northeastern Poland within the framework of our monitoring program. The compounds chosen were the most frequently detected in strawberries (Łozowicka et al. 2012a, b, 2013).

Multivariate statistical analyses are often used in data analysis and include many methods such as principal component analysis (PCA), multivariate curve resolution (MCR), partial least squares (PLS), clustering (K-Means) and soft independent modeling of class analogies (SIMCA). One of the techniques for finding patterns, correlations and identifying the relationship between many variables is PCA, which has been applied in many different areas. PCA is most frequently used by researchers for chemical process modelling and monitoring. For example, de Sousa et al. (2014) detected correlations between the maturation parameters of tomato fruits and the matrix effect of pesticides, and Javorekova et al. (2010) compared changes in the microbial composition of soil samples from different sites and treatments. However, there is lack of research on the application of PCA for quantifying the relationship between the effectiveness of processing methods and the properties of pesticides with risk assessment studies.

Therefore, the first objective of this study was to evaluate the efficiency of four processing techniques for reducing the concentrations of a wide spectrum of the most commonly detected pesticides in strawberries and to determine the processing factors for a particular combination pesticide/processing technique/matrix. The second aim was to use the calculated PFs for a dietary risk assessment. The third goal was to determine correlations between the selected properties of 16 pesticides and their removal efficiency, expressed as PFs, obtained in each process using a PCA.

## Materials and methods

### Field trials

Strawberries (variety *Senga Sengana*) were collected from open field trials conducted in the experimental fields located in Nowy Dwór (in northeastern Poland (Podlasie)) with no previous pesticide applications. The field (approximately 90 m^2^) was divided into 6 m^2^ plots, and the following fungicides and insecticides were applied (each plant protection product on one plot): boscalid, pyraclostrobin (Signum 33 WG); bupirimate (Nimrod 250 EC); cyprodinil, fludioxonil (Switch 62.5 WG); fenhexamid (Teldor 500 SC); folpet (Folpan 80 WG); iprodione (Rovral Aquaflo 500 SC); tetraconazole (Domark 100 EC); trifloxystrobin (Zato 50 WG); acetamiprid (Mospilan 20 SP); alpha-cypermethrin (Fastac 100 EC); chlorpyrifos (Dursban 480 EC); deltamethrin (Decis 2.5 EC); lambda-cyhalothrin (Karate Zeon 050 CS); and pirimicarb (Pirimor 500 WG).

Strawberries were sprayed on plants (at crop stage BBCH code 81 = maturity of fruit, beginning of ripening: most fruits in white colour) using these plant protection products (PPP) at twice the recommended dose (Polish Ministry of Agriculture website) by a specialized operator using a backpack sprayer at normal settings and timing to ensure a sufficient primary pesticide deposit for subsequent processing. The total rainfall was 58.4 L/m^2^, and the daily maximum, minimum and medium temperatures were 25, 13 and 19 °C, respectively, from the day of pesticide application until harvest.

### Samples

Strawberry samples were collected after harvest interval of the PPPs. Fruits were randomly collected to obtain 10 kg of strawberries, packed in polyethylene bags, transported under refrigerated conditions to the laboratory and stored in a refrigerator at 4 °C prior to analysis to the next day.

### Sample preparation and processing

**Fig. 1:**
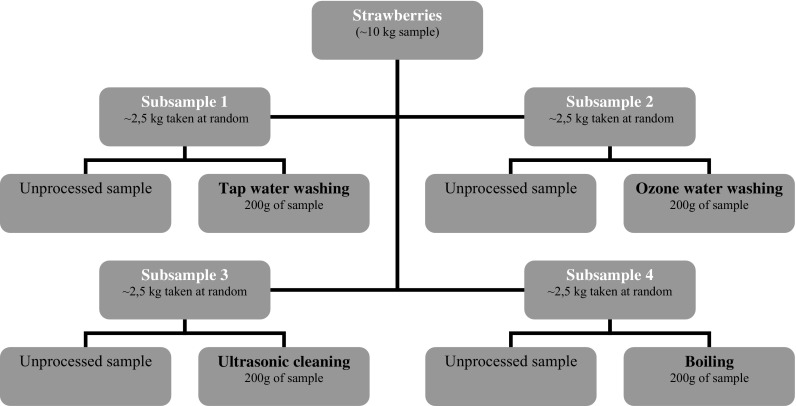
Sampling and processing scheme

From approximately 10 kg of samples taken from each plot, four representative analytical subsamples were separated (approximately 2.5 kg each). To minimize the factor of variability, fruit was chosen randomly and each treatment was performed in triplicate. Figure 1 shows the sampling scheme and processing procedure.

#### Overview of processing and sampling steps

As shown in Fig. 1, each analytical subsample was divided into two parts. One part (0.5 kg collected randomly) was not subject to any processing (unprocessed sample). This part of the subsample was homogenized in a Waring blender (Waring Laboratory Science, Stamford CT, USA) and frozen until analysis. The second part (the remaining 2 kg collected randomly) was divided into 200 g samples and processed for 1, 2 or 5 min. After processing, the samples were air-dried under a fume hood under ambient conditions for 5 min. Immediately after processing, all of these samples were blended and deep-frozen (with liquid nitrogen freezing) in polypropylene bags (−20 °C) until analysis.

#### Unprocessed strawberries

The strawberries which did not undergo any processing were used to evaluate the initial concentration of pesticides which were essential to calculate processing factors.

#### Tap water washing

Fruit samples of 200 g were washed using 1 L chlorinated, tap water (20 °C, 0.1 mg Cl_2_/L) by immersion for 1, 2 and 5 min.

#### Ozone water washing

Ozone was generated by a GL-2186 ozone generator (WRC Multiozon, Poland) at a flow rate of 1 L/min. The maximum amount of ozone that could be dissolved under these conditions was 1 mg O_3_/L. Then, 200 g of fruits were immersed in this solution (20 °C, 1 mg O_3_/L) for 1, 2 and 5 min.

#### Ultrasonic cleaning

Fruit samples of 200 g were placed into a stainless steel basket, soaked in 1 L of tap water and cleaned in an ultrasonic cleaner (Polsonic Sonic 6, Poland, frequency 40 kHz, power 2 × 240W peak/period) for 1, 2 and 5 min.

#### Boiling

Fruit samples of 200 g were put into a stainless steel basket and placed into 1 L of boiling water (100 °C) for 1, 2 and 5 min.

### Calculation of processing factors

Processing factors were calculated for all processing treatments as shown below (Eq. (1)), where *C* is the concentration:

1 $$ \mathrm{P}\mathrm{F}=\frac{C_{\mathrm{residues}\kern0.5em \mathrm{in}\kern0.5em \mathrm{proceed}\kern0.5em \mathrm{commodity}\kern0.5em \left(\mathrm{mg}/\mathrm{kg}\right)}}{C_{\mathrm{residues}\kern0.5em \mathrm{in}\kern0.5em \mathrm{raw}\kern0.5em \mathrm{commodity}\kern0.5em \left(\mathrm{mg}/\mathrm{kg}\right)}} $$

- PF < 1: reduction factor commodity
- PF > 1: concentration factor of pesticide residue in the processed commodity

### Reagents and materials

The analytical standards of pesticides were purchased from Dr. Ehrenstorfer GmbH (Augsburg, Germany) (purity above 95 %). Acetone, n-hexane and diethyl ether were of pesticide residue grade and were obtained from J.T. Baker (Deventer, Holland). Florisil (60–100 mesh) was supplied by J.T. Baker (Deventer, Holland) and anhydrous sodium sulphate by Fluka (Seelze-Hannover, Germany). Silica gel was obtained from Merck (Darmstadt, Germany).

### Extraction procedure

**Fig. 2:**
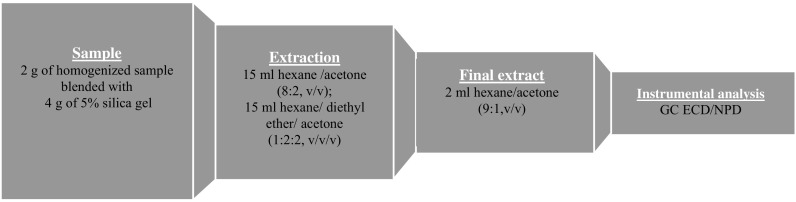
Scheme of MSPD sample preparation

Strawberry samples were prepared according to the original matrix solid-phase dispersion method (MSPD) described in a previous study (Łozowicka et al. 2009). The scheme of the extraction procedure is presented in Fig. 2. The extraction included several steps as follows: (i) 2.0 g of sample with 4.0 g of Florisil was put into a mortar, blended with a pestle and transferred to the glass column with anhydrous sodium sulphate (5.0 g) and silica gel (2.5 g); (ii) 15 mL of each solvent mixture: hexane/acetone (8:2) and hexane/diethyl ether/acetone (1:2:2, *v*/*v*/*v*) was added and evaporated to dryness (40 °C); (iii) then dissolved in 2 mL hexane/acetone (9:1) and the final extract was transferred into a gas chromatography (GC) vial.

### Gas chromatography analysis

GC was conducted using an Agilent 7890 A series gas chromatograph (Waldbronn, Germany) equipped with a nitrogen-phosphorous (NP) and an electron capture (EC) detector. Chromatographic separation was achieved on an HP-5 column (30 m × 0.32 mm, film thickness 0.50 μm). Two microlitres of the final sample extract was injected at 210 °C in splitless mode (purge-off time 2 min). The operating conditions were as follows: detectors and injector temperature, 210 °C; carrier gas, helium at a flow rate of 3.0 mL/min; detector temperature, 300 °C (EC and NP); make-up gas, nitrogen at a flow rate of 57 mL/min (EC) and 8 mL/min (NP) hydrogen at 3.0 mL/min, air at 60 mL/min; and oven initial temperature, 120 °C, increased to 190 °C at 16 °C/min, then to 230 °C at 8 °C/min and finally to 285 °C at 18 °C/min and held for 10 min (EC and NP). The total time of analysis was 20 min.

### Risk assessment with and without the correction for PFs

In this study, an acute (short-term) risk assessment model was used. Calculated PFs were used for assessment of acute risk exposure without a correction for PF (Eq. (2)) and with the correction for PF (Eq. (3)). The risk for active substances with residue concentrations exceeding the MRL was calculated and compared with the ARfD (acute reference dose).

The acute dietary consumer exposure to pesticide residues was estimated by using a calculation model developed by EFSA (PRIMo, Pesticide Residue Intake Model, revision 2) for two sub-populations, children (2–4 years of age) and adults (14–80 years of age), accepting consumption at the level of the 97.5 percentile. Two different versions of the equation (without correction for PF using Eq. (2) and with correction using Eq. (3)) for the calculation of the international estimated short-term intake (IESTI) were used:

2 $$ \mathrm{IESTI}=\frac{\mathrm{LP}*\left(\mathrm{H}\mathrm{R}\right)*v}{\mathrm{bw}} $$

3 $$ \mathrm{IESTI}*=\frac{\mathrm{LP}\ast \left(\mathrm{H}\mathrm{R}*\mathrm{P}\mathrm{F}\right)\ast v}{\mathrm{bw}} $$

(in mg/kg bw)

The following definitions apply to these equations:

- LP—highest large portion, in kilogram of food per day;
- HR—highest residue, in gram per kilogram body weight;
- HR*PF—highest residue in the processed commodity, in milligram per kilogram, calculated by multiplying the HR in the raw commodity by the PF;
- v—variability factor; and
- bw—average body weight for a population age group, in kilogram.

The assessment of the acute exposure was based on a worst-case scenario, i.e. consumption data for consumers with extreme food consumption habits were combined with the highest residue concentration.

### Statistical analysis

A principal component analysis (PCA) was performed to explain the correlations between physicochemical parameters, the mode of action and processing factors for the 16 pesticides and to verify the effect of each processing technique. Data were statistically evaluated by PCA using Statistica version 10.0 software (StatSoft).

## Results and discussion

### Recovery studies

Standards of pesticides were used for calibration curves (0.001–5.00 μg/mL) and injected into the GC-NPD/ECD system under the conditions stated in the GC analysis section. The linearity of the method was evaluated within the range of 0.01–5.00 μg/mL, with correlation coefficients (*R*^2^ > 0.995). The limit of quantification (LOQ) was determined with a signal-to-noise ratio (S/N) = 10, and the limit of detection (LOD) (S/N) = 3. LODs were below 0.003 mg/kg for all 16 analysed pesticides, and LOQs were at 0.01 mg/kg. The validation was performed for five replications at three different spiking levels (i.e. 0.01, 0.30 and 3.00 mg/kg). In all cases, the results of recovery tests were acceptable according to the validation and quality control criteria for pesticide residue analysis established by the European Commission (2014 SANCO/12571/2013) and average recoveries ranged from 78.5 to 101.0 %, with a maximum relative standard deviation (RSD) of 12.0 % (Table 1).

**Table 1:** Mean recoveries and relative standard deviation (RSD) in strawberries at three spiking levels

Nr	Pesticide	Spiking level		
		0.01 mg/kg	0.30 mg/kg	3.00 mg/kg
		Recovery (±RSD)	Recovery (±RSD)	Recovery (±RSD)
1	Acetamiprid	79.2 (±5.4)	98.5 (9.8)	84.2 (±9.0)
2	Alpha-cypermethrin	90.0 (±7.3)	92.2 (7.7)	96.0 (±10.2)
3	Boscalid	100.2 (±4.7)	91.3 (6.6)	93.5 (±12.0)
4	Bupirimate	85.6 (±7.9)	99.5 (8.1)	82.4 (±6.2)
5	Chlorpyrifos	94.2 (±5.8)	94.2 (3.9)	86.2 (±4.6)
6	Cyprodinil	79.9 (±9.5)	98.5 (4.8)	85.1 (±9.3)
7	Deltamethrin	92.1 (±4.4)	82.1 (5.0)	79.6 (±7.2)
8	Fenhexamid	97.5 (±6.4)	98.5 (6.2)	87.0 (±6.4)
9	Fludioxonil	84.9 (±8.7)	82.7 (4.3)	93.1 (±2.9)
10	Folpet	78.5 (±10.4)	98.5 (8.4)	101.0 (±12.0)
11	Iprodione	88.9 (±7.8)	80.6 (9.7)	100.3 (±5.5)
12	Lambda-cyhalothrin	78.7 (±6.1)	82.3 (9.6)	101.0 (±7.7)
13	Pirimicarb	92.3 (±5.4)	89.5 (9.4)	95.7 (±6.3)
14	Pyraclostrobin	81.5 (±6.3)	97.9 (6.2)	99.0 (±4.9)
15	Tetraconazole	89.7 (±8.5)	95.5 (5.6)	89.2 (±6.5)
16	Trifloxystrobin	81.4 (±7.8)	91.2 (7.0)	83.5 (±8.0)

### Unprocessed strawberries

The unprocessed, raw strawberry samples were used to calculate the PFs, and these values describe the efficiency of reducing the pesticide residue level in food processing. Concentrations of the pesticides analyses in unprocessed samples are summarized in Table 2.

**Table 2:** Effect of processing on pesticide residues in strawberry

Nr	Active substance	Treatment	**Time** 1 min		Time 2 min		**Time** 5 min	
			Mean concentration (mg/kg) (±SD)	PF	Mean concentration (mg/kg) (±SD)	PF	Mean concentration (mg/kg) (±SD)	PF
Fungicides								
1	Boscalid	Raw	0.354 (±0.089)	–	–	–	–	–
	MRL	A	0.237 (±0.112)	0.67	0.222 (±0.104)	0.63	0.215 (±0.098)	0.61
	EU = 10.0 mg/kg	B	0.174 (±0.098)	0.49	0.131 (±0.153)	0.37	0.131 (±0.067)	0.37
		C	0.228 (±0.103)	0.64	0.132 (±0.071)	0.37	0.115 (±0.066)	0.32
		D	0.281 (±0.145)	0.79	0.203 (±0.096)	0.57	0.144 (±0.077)	0.41
2	Bupirimate	Raw	0.122 (±0.034)	–	–	–	–	–
	MRL	A	0.114 (±0.052)	0.94	0.102 (±0.048)	0.84	0.098 (±0.049)	0.80
	EU = 1.0 mg/kg	B	0.084 (±0.039)	0.69	0.074 (±0.029)	0.61	0.068 (±0.033)	0.56
		C	0.064 (±0.035)	0.53	0.054 (±0.027)	0.44	0.053 (±0.028)	0.43
		D	0.071 (±0.033)	0.59	0.069 (±0.036)	0.57	0.047 (±0.024)	0.39
3	Cyprodinil	Raw	0.270 (±0.120)	–	–	–	–	–
	MRL	A	0.229 (±0.121)	0.85	0.179 (±0.096)	0.66	0.124 (±0.062)	0.46
	EU = 5.0 mg/kg	B	0.242 (±0.109)	0.90	0.143 (±0.071)	0.53	0.133 (±0.071)	0.49
		C	0.164 (±0.083)	0.61	0.142 (±0.069)	0.53	0.130 (±0.058)	0.48
		D	0.209 (±0.097)	0.77	0.179 (±0.088)	0.66	0.154 (±0.066)	0.57
4	Fenhexamid	Raw	0.444 (±0.145)	–	–	–	–	–
	MRL	A	0.323 (±0.162)	0.73	0.298 (±0.152)	0.67	0.190 (±0.097)	0.43
	EU = 5.0 mg/kg	B	0.366 (±0.184)	0.83	0.363 (±0.181)	0.82	0.223 (±0.116)	0.50
		C	0.371 (±0.175)	0.84	0.360 (±0.170)	0.81	0.244 (±0.111)	0.55
		D	0.316 (±0.157)	0.71	0.223 (±0.116)	0.50	0.229 (±0.105)	0.52
5	Fludioxonil	Raw	0.104 (±0.050)	–	–	–	–	–
	MRL	A	0.091 (±0.045)	0.88	0.064 (±0.031)	0.62	0.049 (±0.034)	0.47
	EU = 4.0 mg/kg	B	0.078 (±0.039)	0.75	0.054 (±0.027)	0.52	0.046 (±0.022)	0.45
		C	0.075 (±0.029)	0.73	0.071 (±0.033)	0.69	0.041 (±0.019)	0.40
		D	0.081 (±0.038)	0.78	0.073 (±0.036)	0.71	0.049 (±0.023)	0.48
6	Folpet	Raw	2.324 (±1.150)	–	–	–	–	–
	MRL	A	2.086 (±1.124)	0.90	1.573 (±0.788)	0.68	1.545 (±0.744)	0.66
	EU = 3.0 mg/kg	B	1.757 (±0.896)	0.76	1.360 (±0.654)	0.59	1.057 (±0.568)	0.45
		C	1.397 (±0.697)	0.60	1.163 (±0.541)	0.50	0.782 (±0.357)	0.34
		D	1.289 (±0.652)	0.55	0.792 (±0.339)	0.34	0.658 (±0.322)	0.28
7	Iprodione	Raw	1.336 (±0.283)	–	–	–	–	–
	MRL	A	1.084 (±0.601)	0.81	1.015 (±0.597)	0.76	0.810 (±0.402)	0.61
	EU = 15.0 mg/kg	B	1.035 (±0.587)	0.77	0.809 (±0.455)	0.61	0.750 (±0.384)	0.56
		C	1.056 (±0.504)	0.79	0.722 (±0.321)	0.54	0.467 (±0.207)	0.35
		D	1.007 (±0.477)	0.75	0.782 (±0.386)	0.59	0.529 (±0.214)	0.40
8	Pyraclostrobin	Raw	0.905 (±0.179)	–	–	–	–	–
	MRL	A	0.720 (±0.361)	0.80	0.670 (±0.324)	0.74	0.620 (±0.325)	0.69
	EU = 1.5 mg/kg	B	0.500 (±0.243)	0.55	0.480 (±0.244)	0.53	0.390 (±0.178)	0.43
		C	0.169 (±0.087)	0.19	0.153 (±0.079)	0.17	0.096 (±0.041)	0.11
		D	0.109 (±0.058)	0.12	0.070 (±0.038)	0.08	0.064 (±0.033)	0.07
9	Tetraconazole	Raw	0.478 (±0.130)	–	–	–	–	–
	MRL	A	0.470 (±0.214)	0.98	0.410 (±0.201)	0.86	0.370 (±0.184)	0.77
	EU = 0.2 mg/kg	B	0.414 (±0.198)	0.86	0.373 (±0.186)	0.79	0.306 (±0.152)	0.64
		C	0.195 (±0.097)	0.41	0.184 (±0.089)	0.53	0.074 (±0.031)	0.15
		D	0.350 (±0.175)	0.73	0.259 (±0.114)	0.63	0.245 (±0.122)	0.51
10	Trifloxystrobin	Raw	0.575 (±0.146)	–	–	–	–	–
	MRL	A	0.510 (±0.211)	0.89	0.460 (±0.198)	0.80	0.280 (±0.196)	0.49
	EU = 1.0 mg/kg	B	0.500 (±0.205)	0.87	0.370 (±0.165)	0.64	0.320 (±0.164)	0.56
		C	0.380 (±0.184)	0.66	0.372 (±0.197)	0.65	0.274 (±0.153)	0.48
		D	0.512 (±0.219)	0.89	0.455 (±0.213)	0.79	0.301 (±0.114)	0.52
Insecticides								
11	Acetamiprid	Raw	0.276 (±0.010)	–	–	–	–	–
	MRL	A	0.210 (±0.095)	0.76	0.180 (±0.097)	0.65	0.120 (±0.058)	0.43
	EU = 0.5 mg/kg	B	0.125 (±0.067)	0.45	0.113 (±0.056)	0.41	0.103 (±0.052)	0.37
		C	0.140 (±0.069)	0.51	0.130 (±0.071)	0.47	0.122 (±0.066)	0.44
		D	0.142 (±0.071)	0.51	0.119 (±0.064)	0.43	0.090 (±0.046)	0.33
12	alpha-Cypermethrin	Raw	0.170 (±0.067)	–	–	–	–	–
	MRL	A	0.110 (±0.052)	0.65	0.090 (±0.044)	0.53	0.079 (±0.037)	0.47
	EU = 0.07 mg/kg	B	0.106 (±0.048)	0.63	0.077 (±0.036)	0.45	0.071 (±0.033)	0.42
		C	0.035 (±0.014)	0.21	0.022 (±0.010)	0.13	0.015 (±0.007)	0.09
		D	0.172 (±0.086)	*1.02*	0.281 (±0.139)	*1.66*	0.298 (±0.156)	*1.76*
13	Chlorpyrifos	Raw	0.100 (±0.071)	–	–	–	–	–
	MRL	A	0.054 (±0.025)	0.54	0.052 (±0.022)	0.52	0.032 (±0.014)	0.32
	EU = 0.2 mg/kg	B	0.045 (±0.019)	0.45	0.034 (±0.018)	0.34	0.025 (±0.011)	0.25
		C	0.058 (±0.026)	0.58	0.050 (±0.023)	0.50	0.021 (±0.019)	0.21
		D	0.080 (±0.041)	0.80	0.051 (±0.026)	0.51	0.047 (±0.012)	0.47
14	Deltamethrin	Raw	0.150 (±0.042)	–	–	–	–	–
	MRL	A	0.130 (±0.062)	0.86	0.120 (±0.061)	0.80	0.110 (±0.054)	0.73
	EU = 0.2 mg/kg	B	0.081 (±0.037)	0.54	0.069 (±0.034)	0.46	0.057 (±0.031)	0.38
		C	0.094 (±0.043)	0.62	0.056 (±0.022)	0.37	0.043 (±0.012)	0.28
		D	0.155 (±0.075)	*1.03*	0.175 (±0.085)	*1.16*	0.198 (±0.099)	*1.32*
15	lambda-Cyhalothrin	Raw	0.277 (±0.013)	–	–	–	–	–
	MRL	A	0.250 (±0.124)	0.90	0.240 (±0.123)	0.86	0.190 (±0.094)	0.68
	EU = 0.5 mg/kg	B	0.162 (±0.088)	0.58	0.262 (±0.140)	0.94	0.152 (±0.077)	0.55
		C	0.131 (±0.063)	0.47	0.127 (±0.067)	0.46	0.115 (±0.059)	0.42
		D	0.330 (±0.152)	*1.19*	0.383 (±0.195)	*1.38*	0.473 (±0.208)	*1.70*
16	Pirimicarb	Raw	0.957 (±0.032)	–	–	–	–	–
	MRL	A	0.820 (±0.413)	0.86	0.810 (±0.401)	0.85	0.760 (±0.389)	0.79
	EU = 3.0 mg/kg	B	0.674 (±0.346)	0.70	0.480 (±0.222)	0.50	0.384 (±0.142)	0.40
		C	0.365 (±0.033)	0.38	0.357 (±0.028)	0.37	0.333 (±0.014)	0.35
		D	0.413 (±0.203)	0.43	0.424 (±0.216)	0.44	0.311 (±0.158)	0.32

PF < 1 reduction factor, PF > 1concentration factor (in italics)

*Raw* unprocessed samples (*n* = 4); Processed samples (*n* = 3): *A* washing with tap water, *B* washing with ozonated water, *C* ultrasonic cleaning and *D* boiling; *SD* relative standard deviation

### Physicochemical properties and mode of action of studied pesticides

The main physical and chemical properties of the studied pesticides, including octanol-water partition coefficient (log*P*), solubility in water (S_w_), boiling point and molecular mass (M) and the mode of action, are presented in Table 3 and discussed individually.

**Table 3:** Physicochemical parameters and mode of action of fungicides and insecticides

Nr	Pesticide	Group	Mode of action	log*P*	*S*_w_	Boiling point (°C)	*M*
1	Acetamiprid	Neonicotinoid	sys	0.8	2950	352.4	222.67
2	alpha-Cypermethrin	Pyrethroid	non-sys	5.5	0.004	826.0	416.30
3	Boscalid	Carboxamide	sys	2.96	4.6	447.7	343.21
4	Bupirimate	Pyrimidinol	sys	3.68	13.06	463.2	316.42
5	Chlorpyrifos	Organophosphate	non-sys	4.7	1.05	395.8	350.89
6	Cyprodinil	Anilinopyrimidine	sys	4	13	405.985	225.29
7	Deltamethrin	Pyrethroid	non-sys	4.6	0.0002	535.8	505.2
8	Fenhexamid	Hydroxyanilide	non-sys	3.51	20	457.9	302.20
9	Fludioxonil	Phenylpyrrole	non-sys	4.12	1.8	420.4	248.19
10	Folpet	Phthalimide	non-sys	3.02	0.8	333.8	296.56
11	Iprodione	Dicarboximide	non-sys	3.1	12.2	not available	330.17
12	lambda-Cyhalothrin	Pyrethroid	non-sys	6.9	0.005	498.9	449.85
13	Pirimicarb	Carbamate	sys	1.7	3100	373.4	238.39
14	Pyraclostrobin	Strobilurin	sys	3.99	1.9	501.1	412.87
15	Tetraconazole	Triazole	sys	3.56	156.6	438.4	372.15
16	Trifloxystrobin	Strobilurin	sys	4.5	0.61	470.345	408.37

Boiling point (°C) at 760 mmHg

*sys* systemic pesticide, *non-sys* non-systemic pesticide, *logP* octanol-water partition coefficient at pH 7, 20 °C, *S*_*w*_ solubility in water at 20 °C (mg/L), *M* molecular mass (g/mol)

### Effect of processing

**Fig. 3:**
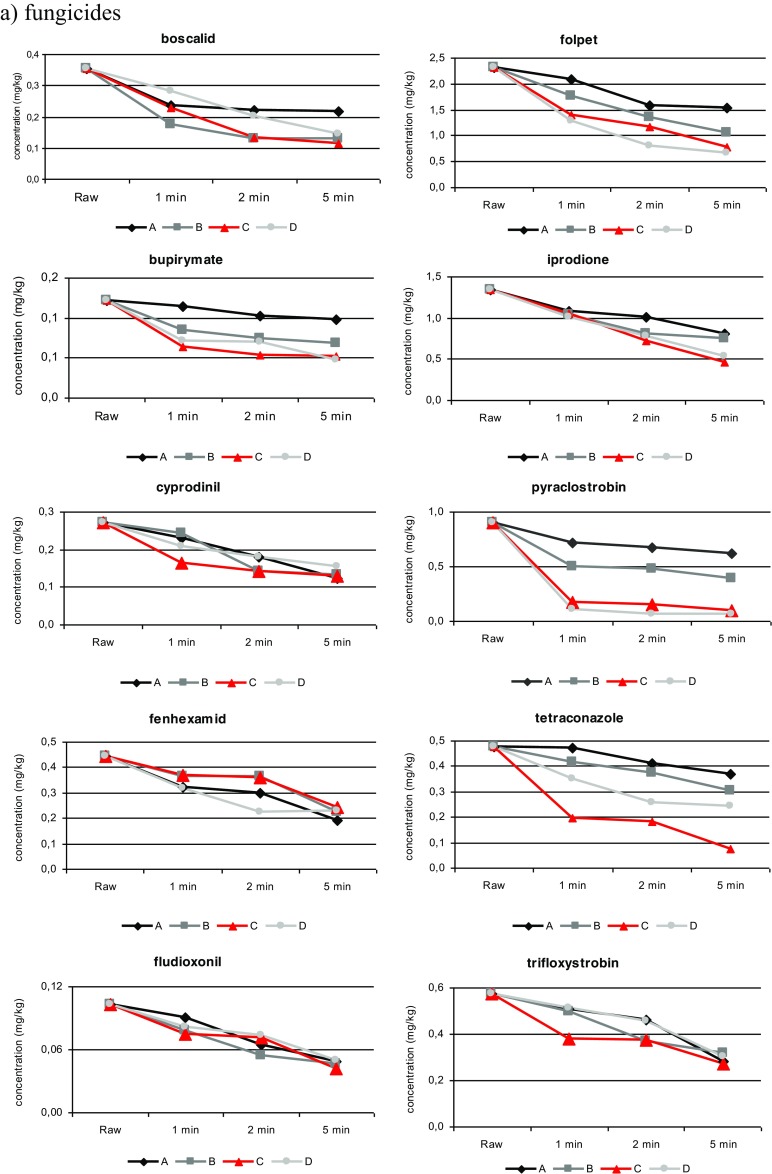

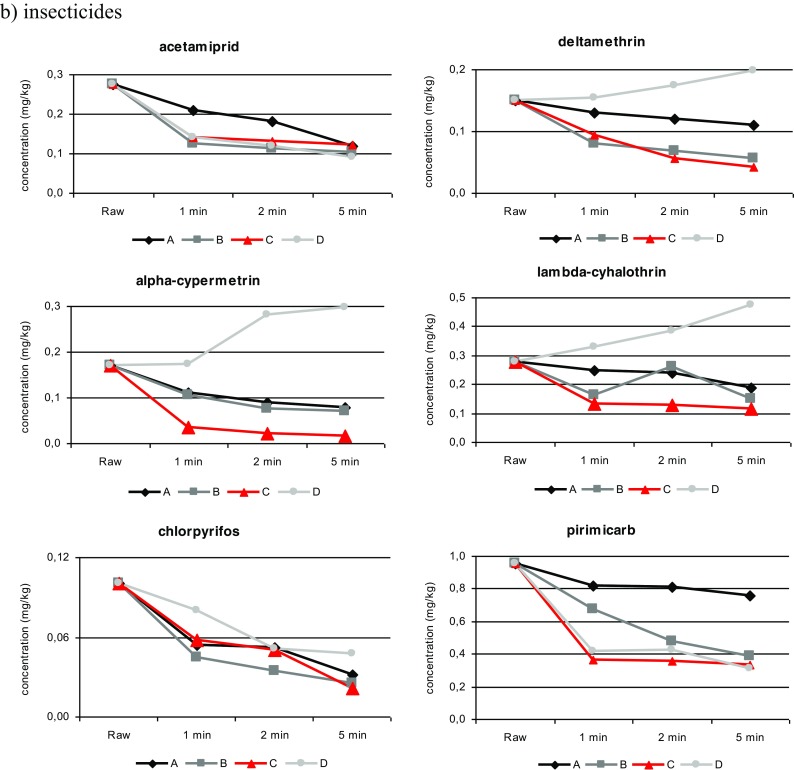
**a**, **b** Pesticide behaviour during processing (*A* washing tap water, *B* washing ozone water, *C* ultrasonic cleaning and *D* boiling)

The processing conditions corresponded as closely as possible to actual conditions that are common in household and industrial practices. All processing methods were conducted over 1, 2 and 5 min, and the change of concentration level over time was analysed. A gradual reduction in the level of nearly all pesticides was noted when the time was increased to 5 min. The concentration changes of the fungicide and insecticide residue levels during processing (Fig. 3a, b) are discussed below for each individual treatment.

To evaluate the effects of processing on pesticide residues in strawberries, processing factors (PFs) related to each process were determined. Processing factors were generally below 1 for most of the studied pesticides, and only after the boiling process did three insecticides exhibit PFs above 1. The behaviour of fungicide and insecticide residue levels during processing is shown in Table 2.

#### Effects of washing with tap water (A)

Washing is the most common form of processing and is a preliminary step in both household and commercial preparations. In this study, washing was done in tap water. The effects of 1, 2 and 5 min of washing with tap water on pesticide residues in strawberries are shown in Table 2. Concentration changes for 16 pesticide residues were observed after 1, 2, and 5 min of treatment. The effectiveness of this process resulted in a 19.8 % reduction for bupirimate and 68.1 % for chlorpyrifos, with PF = 0.80 and PF = 0.32 (for 5 min), respectively. Washing with tap water significantly reduced (over 50 %) the concentrations of three insecticides and two fungicides. A gradual reduction was noted when the time was increased to 5 min for acetamiprid, alpha-cypermethrin, chlorpyrifos, cyprodinil and fenhexamid by 56.5, 53.4, 68.1, 54.1, and 57.2 %, respectively. These data are consistent with other studies conducted on raw cucumbers (Liang et al. 2012), where increasing the time of the washing process yielded a lower PF.

Our results can be explained through the analysis of the relationship between the physicochemical properties of the studied pesticides, including their solubility in water, octanol-water partition coefficient and PF values. Polar, water-soluble pesticides are more readily removed than low-polarity materials (Holland et al. 1994). A number of studies have reported that pesticides with a lower octanol-water partition coefficient are more easily removed by washing (Kong et al. 2012; Zhao et al. 2014). In our study, acetamiprid, with a low log*P* = 0.8 and high solubility in water 2950 mg/L, exhibited a low PF = 0.43, in contrast with deltamethrin (log*P* = 4.6, *S*_w_ = 0.0002 mg/L) PF = 0.73 (Table 2). As discussed above, the log*P* and solubility were the key factors affecting the reduction.

We can presume that a high solubility in water does not have an influence on the effectiveness of washing in every case, but the removal of residues also depends on the location of this substance in the plant material during transpiration. Thus, pesticides such as bupirimate and pirimicarb (with a systemic mode of action) are less likely to be transported into the internal parts of strawberries, despite their high water solubility, and thus, they exhibited PF = 0.80 and PF = 0.79, respectively.

#### Effects of washing with ozonated water (B)

Ozone (O_3_) is one of the most potent sanitizers against a wide spectrum of microorganisms (Khadre et al. 2001) and is considered to be the most suitable for removing pesticide residues from fruits and vegetables and for controlling microbes of food safety concern (Gabler et al. 2010). In our study, after 5 min of washing in ozone water, pesticide residues were reduced by between 36.1 % (PF = 0.64) for tetraconazole and 75.1 % (PF = 0.25) for chlorpyrifos (Table 2).

As in the case of washing with chlorinated water, the highest reduction was also observed for chlorpyrifos (Table 2). Chlorpyrifos is a non-systemic insecticide, acting only when it comes into direct contact with plant tissues, and is not transported to other plant parts; therefore, its residues were amenable to simple processing operations, and a larger decrease was expected. In contrast, systemic agents such as tetraconazole or bupirimate, which penetrate to deeper tissue layers of the strawberries, were more difficult to remove (PFs ≥ 0.56).

Comparing the above results, ozonated water was more effective than tap water in pesticide removal. Chen et al. (2013) concluded that removal efficiency increased when vegetables were treated with ozone, and our results confirmed this hypothesis.

Such a large reduction is possible because the dissolved ozone generates hydroxyl radicals that are highly effective at decomposing organic molecules such as pesticide residues (Sumikura et al. 2007; Takahashi et al. 2003).

Moreover, we can conclude that the molecular weight of each compound could affect the percentage of reduction. Washing with ozone water was more effective in the removal of pesticides with a lower molecular mass, such as boscalid or acetamiprid (*M* ≤ 343.21 g/mol) (both have PF = 0.37), compared with tetraconazole (*M* = 372.15 g/mol) and trifloxystrobin (*M* = 408.37 g/mol) (with PF = 0.64 and PF = 0.56, respectively (Table 2)).

#### Effects of ultrasonic cleaning (C)

Pesticide residues were eliminated most effectively by ultrasonic cleaning. The concentration levels of pesticide residues were strongly changed in this process. As shown in Table 2, pesticide reduction increased with time. The efficiency of ultrasonic cleaning after 5 min ranged between 45.1 % (fenhexamid, PF = 0.55) and 91.2 % (alpha-cypermethrin, PF = 0.09), and PFs were below 0.55 for all active substances.

High reductions, above 70 %, were also found for pyraclostrobin (89.4 %), tetraconazole (84.5 %) and chlorpyrifos (79.1 %) with PFs 0.11, 0.15 and 0.21, respectively (Table 2). Ultrasonic cleaning reduced those pesticide residues to a greater extent than did tap water soaking, because cavitation bubbles created many small air bubbles in the liquid. These bubbles grew, expanded and regularly broke out violently, which generated mechanical energy in the form of shockwaves and caused distribution within the very small pores on the asymmetric surfaces of strawberries. Thus, pesticide residue reduction was more efficient than cleaning without the ultrasonic cleaner.

We can assume that the effectiveness of ultrasonic cleaning also depended on the mode of action of the studied compounds. As reported in Table 2, the non-systemic pesticides (alpha-cypermethrin, chlorpyrifos, deltamethrin, folpet and iprodione) were easily removed by ultrasonication (PF ≤ 0.35), compared with bupirimate, acetamiprid, trifloxystrobin and cyprodinil (0.43 ≤ PF ≤ 0.48), which have systemic modes of action. Ultrasonic cleaning has rarely been studied, but our results confirmed that it is an attractive technique for the removal of pesticide residues in industry.

#### Effects of boiling (D)

Boiling is a method of cooking food using a high water temperature and is often used to make strawberry preserves. The concentrations of the studied pesticides were highly reduced using this process. Boiling for 5 min caused a greater reduction for most pesticides than did both types of washing. Pyraclostrobin showed the highest reduction during this process (92.9 %, PF = 0.07), while cyprodinil showed the lowest (42.8 %, PF = 0.57) (Table 2).

However, the results obtained from the thermal process indicated that the concentrations of three insecticides from the pyrethroid class (alpha-cypermethrin (PF = 1.02, 1.66 and 1.76), deltamethrin (PF = 1.03, 1.16 and 1.32) and lambda-cyhalothrin (PF = 1.19, 1.38 and 1.70)) increased, and processing factors above 1 were noted. These results could be because these pesticides were concentrated as the water evaporated from the strawberries during boiling (Amvrazi 2011). Notably, similar findings were obtained by Rasmusssen et al. (2003)), who found that boiling did not reduce pesticide residues in apples.

The decreases could also be explained by the fact that water-soluble pesticides such as pirimicarb (*S*_w_ = 3100 mg/L) and acetamiprid (*S*_w_ = 2950 mg/L) were significantly eliminated (PFs ≤ 0.33), in contrast with alpha-cypermethrin, deltamethrin and lambda-cyhalothrin (PFs > 1) with low solubilities of 0.004, 0.0002 and 0.005 mg/L, respectively. These results may also be interpreted based on the boiling point of each pesticide (Table 3). As shown in Table 3, compounds with high boiling points, such alpha-cypermethrin, lambda-cyhalothrin or deltamethrin, were barely reduced in comparison with acetamiprid or pirimicarb, which have low boiling points.

The disappearance of pesticide residues during thermal processing could be due to decomposition by the effect of heat, the stronger adsorption of pesticides onto plant tissues and/or the solubility of pesticides in water (Table 3). Processes involving heating can increase volatilization, hydrolysis or other forms of degradation and thus reduce residue levels (Holland et al. 1994).

In addition, each compound has different metabolites, and they are different in different matrices or may be produced under different processing conditions (European Commission 1997a, b). Thus, thermally unstable compounds, such as folpet, were significantly reduced (71.7 %), most likely by the formation of degradation products during boiling; however, these products were not investigated in this study.

### Risk assessment

The potential short-term consumer risk, before and after processing of strawberries, for alpha-cypermethrin was performed for two populations, adults and children. Alpha-cypermethrin, with an MRL = 0.07 mg/kg (EU Pesticide Database), showed more than twice the concentration of the safety limit in raw strawberries when we used a double dose of PPP during our experiments. Alpha-cypermethrin is a non-systemic, broad-spectrum, insecticidal pyrethroid. It acts through digestion in the target organism’s gut and affects the central and peripheral nervous system through sodium channel modulation. Thus, among the analysed pesticides, alpha-cypermethrin has a relatively low value of ARfD (0.04 mg/kg).

Table 4 presents the results of the short-term risk assessment where the ARfD in processed strawberries was below 4 % for adults and 12 % for children (without the correction for PF). Assessment of short-term risk calculated with the correction for PF was lower for both populations than when the PF values were excluded from the calculations with one exception. In boiling, value of PF was above 1 and after correction, IESTI and %ARfD were higher than those obtained without including PF. However, estimated short-term intakes for children and adults, in both cases (without and with correction for PFs), did not exceed the safety limit (100 % ARfD) in all treatments.

**Table 4:** International estimated short-term intakes (IESTI) for alpha-cypermethrin (ARfD = 0.04 mg/kg bw, elaborated by Joint FAO/WHO Meeting Pesticide Residues JMPR 2006)

	Concentration (mg/kg)	PF	*v* = 1	Adults (average bw 70 kg)				Children (average bw 17 kg)			
				HR (g/kg bw)	LP (g/person)	IESTI (mg/kg bw/day)	% ARfD	HR (g/kg bw)	LP (g/person)	IESTI (mg/kg bw/day)	% ARfD
				5.29	333.0			15.59	251.8		
MRL	0.070	–				0.0004	0.9			0.0011	2.7
Raw	0.170	–				0.0009	2.2			0.0027	6.6
A	0.079	0.47				0.0004	1.0			0.0012	3.1
						0.00019*	0.47*			0.00056*	1.4*
B	0.071	0.42				0.0004	0.9			0.0011	2.8
						0.00017*	0.42*			0.00046*	1.15*
C	0.015	0.09				0.0001	0.2			0.0002	0.6
						0.00001*	0.02*			0.00002*	0.05*
D	0.298	1.76				0.0016	3.9			0.0046	11.6
						0.0028*	7.0*			0.0081*	20.2*

*A* washing with tap water, *B* washing with ozonated water, *C* ultrasonic cleaning, *D* boiling

*IESTI and % ARfD with the correction for PF

Both adults and children have similar values of LP (highest large portion is 333.0 and 251.8 g/person, respectively). However, the calculated IESTI was three times higher for children than that for adults without the correction for PF, and almost ten times higher when the PFs were used (IESTI*). This could be because children are a vulnerable group of consumers, who are, due to their lower body weight, exposed to relatively higher pesticide residue levels.

### Correlation of selected parameters of pesticides and removal effectiveness using PCA

In order to better understand the correlation between the selected parameters of the pesticides and removal effectiveness (expressed as processing factors), a principal component analysis (PCA) was carried out. Figure 3 presents the correlation between the processing factors of pesticides and their physicochemical parameters for each treatment. The relationship between the solubility (*S*_w_), polarity (log*P*), and the mode of action (systemic and non-systemic) of the studied pesticides and the effectiveness of technology (PFs) is discussed below, with respect to each water process.

**Fig. 4:**
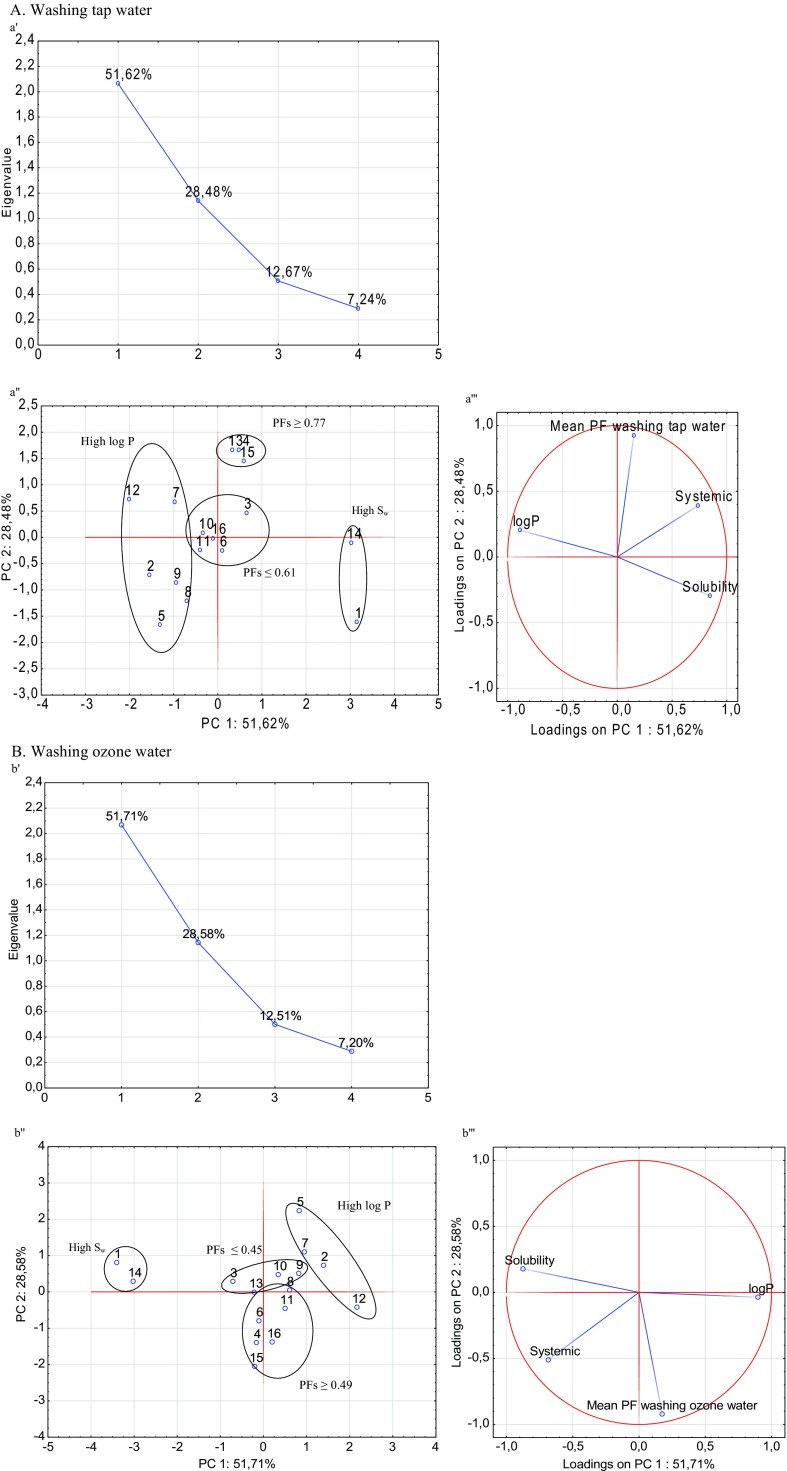

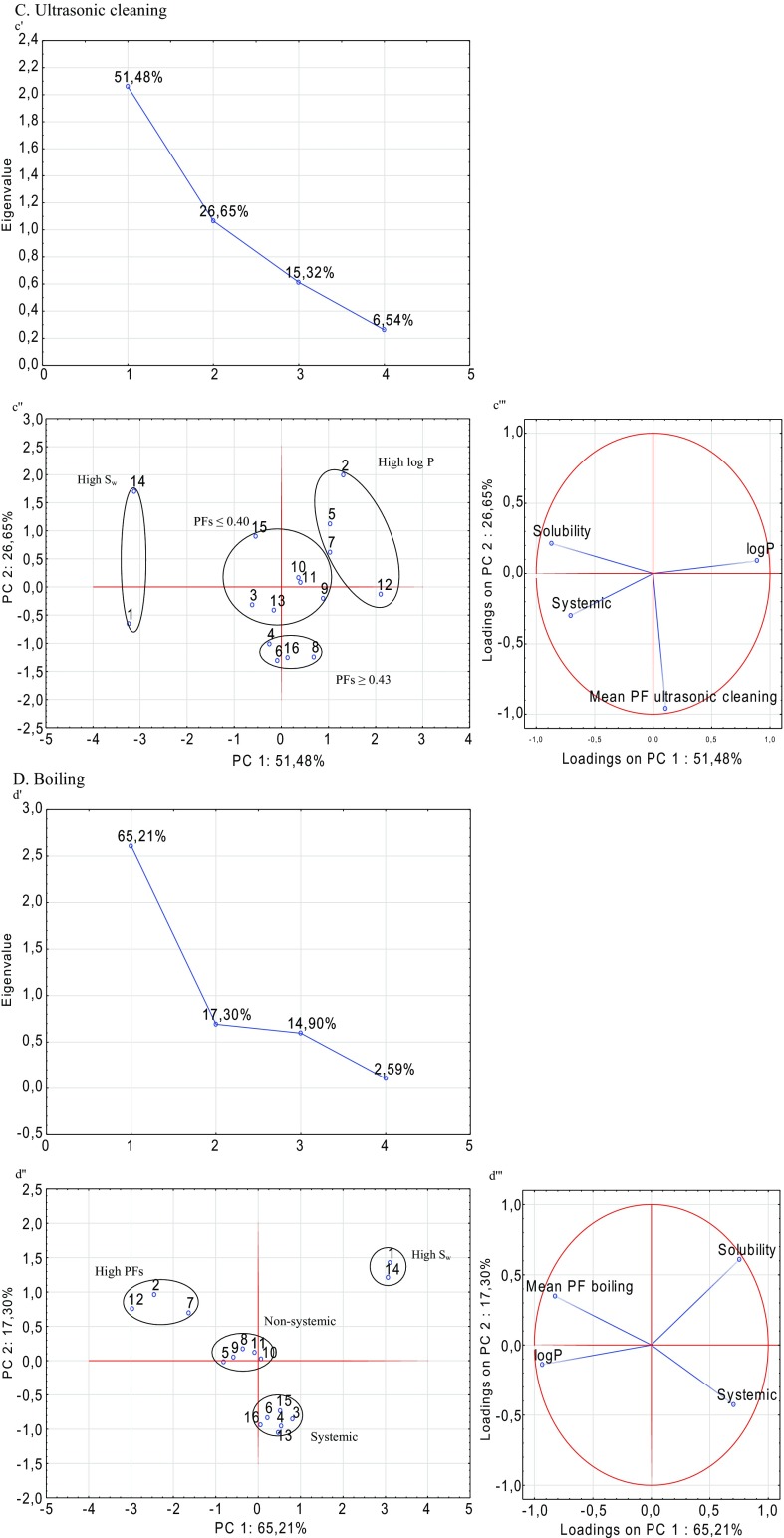
**a**–**d** The scree (*a*'÷*d*'), score (*a*''÷*b*'') and loading (*a*'''÷*d*''') plots of PCA for 16 pesticides

The scree plots obtained in the PCA for each process are shown in Fig. 4a–d. According to the Kaiser’s eigenvalue-greater-than-one rule, the first two principal components (PC) fulfilled this criteria. Thus, the first PC1 and the second principal component PC2 were further analysed.

The PCA analysis revealed a correlation score (Fig. 4a''–d'') and loading (Fig. 4a'''–d''') plots that described more than 78 % of the variation in the first two principal components. The two significant PCs were extracted by covering 80.1 % of the variance in washing with tap water (PC1 51.62 % and PC2 28.48 %) (Fig. 4a), 80.3 % of the variance in washing with ozone water (PC1 51.71 % and PC2 28.58 %) (Fig. 4b), 78.1 % of the variance in ultrasonic cleaning (PC1 51.28 % and PC2 26.65 %) (Fig. 4c) and 82.5 % of the variance in boiling (PC1 65.21 % and PC2 17.30 %) (Fig. 4d).

Interpreting the scores and loadings, the pesticides were categorized, as shown in Fig. 4. Analysed pesticides were grouped into four clusters (Fig. 4a''–b''). The same two groups of compounds were noted in the cases of chlorine, ozone washing and ultrasonic cleaning. The first group was water-soluble pesticides (acetamiprid and pyraclostrobin) and the second was non-polar substances (alpha-cypermethrin, chlorpyrifos, deltamethrin and lambda-cyhalothrin). The dominant variables were solubility and polarity (Fig. 4a'''–d'''). In thermal processing (boiling), the variable with a high contribution was the effectiveness of the treatment and the concentration factors for alpha-cypermethrin, deltamethrin and lambda-cyhalothrin were observed. The results from the PCA confirmed our previous assumptions.

## Conclusions

The studied water technologies, that is, washing with tap and ozone water, supported by ultrasonic waves and boiling were used to determine the effectiveness of the removal of 16 pesticide residues in strawberries. Concentration changes of 16 pesticide residues after 1, 2 and 5 min treatments were observed, and a gradual reduction was noted. The effect of the long treatment time, up to 5 min, had a significant effect on the reduction of several pesticide residues in all procedures.

Processing factors for all pesticides were determined and ranged between 0.07 and 1.76. Washing with tap and ozone water significantly removed pesticide residues present in raw strawberries. Boiling was sufficient for the removal of most pesticide residues with the exception of the pyrethroid class (alpha-cypermethrin, deltamethrin and lambda-cyhalothrin). The concentration factors of those active substances may be the result of water loss during boiling. Ultrasonic cleaning was the most effective procedure for the complete removal of the residues of all studied compounds. Most of processing factors were explained in terms of water solubility, polarity and the penetration mechanism of the studied pesticides.

The results show that water treatments could be useful for the partial removal of several pesticide residues from strawberries under both household and industrial conditions.

To the best of our knowledge, this paper reports for the first time the effectiveness of water technologies for the removal of several pesticide residues from strawberries. The data from this study helps in the estimation of processing factors for 16 pesticides in specific processes. These values will complement the limited databases and aid in risk assessments of processed strawberries. Using PCA, a powerful statistical tool, processing factors of other pesticides in soft fruits can be predicted.

With the growing need to identify food safety hazards, this type of study is required for a more realistic estimation of the dietary intake of the pesticides.

## Abbreviations

PF: Processing factor
IESTI: International estimated short-term intake
PCA: Principal component analysis

## References

[CR1] Adewuyi YG (2001). Sonochemistry: environmental science and engineering applications. Industrial and Engineering Chemistry Research.

[CR2] Aktar MDW, Sengupta D, Chowdhury A (2009). Impact of pesticides use in agriculture: their benefits and hazards. Interdisciplinary Toxicology.

[CR3] Amvrazi, E.G. (2011) Fate of pesticide residues on raw agricultural crops after postharvest storage and food processing to edible portions. pesticides - formulations, effects, fate. http://www.intechopen.com/books/pesticidesformulations-effects-fate/fate-of-pesticide-residues-on-raw-agricultural-crops-after-postharvest-storage-and-food-processing-t. Accessed 2 Dec 2014.

[CR4] Angioni A, Schirra M, Garau VL, Melis M, Tuberos CIG, Cabras P (2004). Residues of azoxystrobin, fenhexamid and pyrimethanil in strawberry following field treatments and the effect of domestic washing. Food Additives and Contaminants.

[CR5] Battino M, Beekwilder J, Denoyes-Rothan B, Laimer M, McDougall GJ, Mezzetti B (2009). Bioactive compounds in berries relevant to human health. Nutrition Reviews.

[CR6] Bonnechère A, Hanot V, Jolie R, Hendrickx M, Bragard C, Bedoret T, van Loco J (2012). Processing factors of several pesticides and degradation products in carrots by household and industrial processing. Journal of Food Research.

[CR7] Bonnechère A, Hanot V, Bragard C, Bedoret T, van Loco J (2012). Effect of household and industrial processing on the levels of pesticide residues and degradation products in melons. Food Additives and Contaminants Part A Chemistry, Analysis, Control, Exposure and Risk Assessment.

[CR8] Bonnechère A, Hanot V, Jolie R, Hendrickx M, Bragard C, Bedoret T, van Loco J (2012). Effect of household and industrial processing on levels of five pesticide residues and two degradation products in spinach. Food Control.

[CR9] Burchat CS, Ripley BD, Leishman PD, Ritcey GM, Y. K, Stephenson GR (1998). The distribution of nine pesticides between the juice and pulp of carrots and tomatoes after home processing. Food Additives and Contaminants.

[CR10] Cabras P, Angioni A, Garau VL, Pirisi FM, Cabitza F, Pala M, Farris GA (2000). Fate of quinoxyfen residues in grapes, wine, and their processing products. Journal of Agriculture and Food Chemistry.

[CR11] Chen JY, Lin YJ, Kuo WC (2013). Pesticide residue removal from vegetables by ozonation. Journal of Food Engineering.

[CR12] Christensen HB, Granby K, Rabølle M (2003). Processing factors and variability of pyrimethanil, fenhexamid and tolylfluanid in strawberries. Food Additives and Contaminants.

[CR13] EU Pesticide Database. http://ec.europa.eu/sanco_pesticides/public/?event=homepage. Accessed 12 Nov 2014

[CR14] European Commission (1997a) Appendix A—metabolism and distribution in plants. 7028/VI/95-rev. 3, 22 July 1997. http://ec.europa.eu/food/plant/protection/resources/publications_en.ht. Accessed 12 Nov 2014

[CR15] European Commission (1997b) Appendix E—processing studies. 7035/VI/95 Rev 5, 22 July 1997. http://ec.europa.eu/food/plant/protection/resources/publications_en.ht. Accessed 12 Nov 2014

[CR16] European Commission (2014). Method validation and quality control procedures for pesticide residues analysis in food and feed. Document No. SANCO /12571/2013. Accessed 2 Sep 2015.

[CR17] Fernandes VC, Domingues VF, Mateus N, Delerue-Matos C (2012). Pesticide residues in Portuguese strawberries grown in 2009–2010 using integrated pest management and organic farming. Environmental Science & Pollution Research.

[CR18] Gabler FM, Smilanick JL, Mansour MF, Karaca H (2010). Influence of fumigation with high concentrations of ozone gas on postharvest gray mold and fungicide residues on table grapes. Postharvest Biology and Technology.

[CR19] Han Y, Xu J, Dong F, Li W, Liu X, Li Y, Kong Z, Zhu Y, Liu N, Zheng Y (2013). The fate of spirotetramat and its metabolite spirotetramat-enol in apple samples during apple cider processing. Food Control.

[CR20] Holland PT, Hamilton D, Ohlin B, Skidmore MW (1994). Effects of storage and processing on pesticide residues in plant products. Pure and Applied Chemistry.

[CR21] Integrated Pest Management (IPM) (2012) Private pesticide applicator training manual 19^th^ Ed. Chapter 1, http://www.extension.umn.edu/agriculture/pesticide-safety/ppat_manual/Chapter%201.pdf

[CR22] JMPR (2006) Pesticide toxicological reference values. http://www.efsa.europa.eu/en/mrls/docs/toxicovaluesr.pdf. Accessed 20 January 2015.

[CR23] Javorekova S, Svrcekova I, Makova J (2010). Influence of benomyl and prometryn on the soil microbial activities and community structures in pasture grasslands of Slovakia. Journal of Environmental Science and Health, Part B.

[CR24] Jurewicz J, Hanke W (2008). Prenatal and childhood exposure to pesticides and neurobehavioral development: review of epidemiological studies. International Journal of Occupational Medicine and Environmental Health.

[CR25] Kaushik G, Satya S, Naik SN (2009). Food processing a tool to pesticide residue dissipation—a review. Food Research International.

[CR26] Keikotlhaile BM, Spanoghe P, Steurbaut W (2010). Effects of food processing on pesticide residues in fruits and vegetables: a meta-analysis approach. Food and Chemical Toxicology.

[CR27] Keikotlhaile, B.M., Spanoghe, P. (2011) Pesticide residues in fruits and vegetables. pesticides—formulations, effects, fate. http://www.intechopen.com/books/pesticides-formulations-effects-fate/pesticide-residues-in-fruits-andvegetables. Accessed 1 Dec 2014

[CR28] Kentish S, Feng H (2014). Applications of power ultrasound in food processing. Annual Reviews of Food Science and Technology.

[CR29] Khadre MA, Yousef AE, Kim JG (2001). Microbial aspects of ozone applications in food: a review. Journal of Food Science.

[CR30] Kim CM, Huat TG (2010). Headspace solid-phase microextraction for the evaluation of pesticide residue contents in cucumber and strawberry after washing treatment. Food Chemistry.

[CR31] Kong ZQ, Dong FS, Xu J, Liu XG, Li J, Li YB (2012). Degradation of acephate and its metabolite methamidophos in rice during processing and storage. Food Control.

[CR32] Krol WJ, Arsenault TL, Pylypiw HM, Mattina MJI (2000). Reduction of pesticide residues on produce by rinsing. Journal of Agricultural.

[CR33] Liang Y, Wang W, Shen Y, Liu Y, Liu XJ (2012). Effects of home preparation on organophosphorus pesticide residues in raw cucumber. Food Chemistry.

[CR34] Łozowicka B, Jankowska M, Kaczyński P (2009). Pesticide residues in Brassica vegetables and exposure assessment of consumers. Food Control.

[CR35] Łozowicka B, Kaczyński P, Jankowska M, Rutkowska E, Hrynko I (2012). Pesticide residues in raspberries (Rubus idaeus L.) and dietary risk assessment. Food Additives and Contaminants: Part B.

[CR36] Łozowicka B, Rutkowska E, Jankowska M, Kaczyński P, Hrynko I (2012). Health risk analysis of pesticide residues in berry fruit from north-eastern Poland. Journal of Fruit and Ornamental Plant Research.

[CR37] Łozowicka B, Kaczyński P, Rutkowska E, Jankowska M, Hrynko I (2013). Evaluation of pesticide residues in fruit from Poland and health risk assessment. Agricultural Science.

[CR38] McDougall, G.J., Stewart, D. (2012) Berries and health: a review of the evidence. Food and health innovation. http://www.northsearegion.eu/files/repository/20131219103705_UK-Enclosure53_sep12.pdf. Accessed 17 Nov 2014

[CR39] Ong KC, Cash JN, Zabik MJ, Siddiq M, Jones AL (1996). Chlorine and ozone washes for pesticide removal from apples and processed apple sauce. Food Chemistry.

[CR40] Polish Ministry of Agriculture web site, The register of plant protection products. http://www.bip.minrol.gov.pl/DesktopDefault.aspx?TabOrgId=647&LangId=0. Accessed 1 Dec 2014

[CR41] Rasmusssen RR, Poulsen ME, Hansen HCB (2003). Distribution of multiple pesticide residues in apple segments after home processing. Food Additives & Contaminants.

[CR42] Rawn D, Quade SC, Sun WF, Fouguet A, Belanger A, Smith M (2008). Captan residue reduction in apples as a result of rinsing and peeling. Food Chemistry.

[CR43] Shabeer ATP, Kaushik B, Manjusha J, Rushali G, Sagar U, Sandip H, Dasharath O (2015). Residue dissipation and processing factor for dimethomorph, famoxadone and cymoxanil during raisin preparation. Food Chemistry.

[CR44] de Sousa FA, Neves AA, de Queiroz MELR, Heleno FF, Teófilo RF, de Pinho GP (2014). Influence of ripening stages of tomatoes in the analysis of pesticides by gas chromatography. Journal of the Brazilian Chemical Society.

[CR45] Sumikura M, Hidaka M, Murakami H, Nobutomo Y, Murakami T (2007). Ozone micro-bubble disinfection method for wastewater reuse system. Water Science and Technology.

[CR46] Takahashi M, Kawamura T, Yamamoto Y, Ohnari H, Himuro S, Shakutsui H (2003). Effect of shrinking microbubble on gas hydrate formation. The Journal of Physical Chemistry B.

[CR47] Wołejko E, Łozowicka B, Kaczyński P (2014). Pesticide residues in berries fruits and juices and the potential risk for consumers. Desalination & Water Treatment.

[CR48] Zhao L, Ge J, Liu F, Jiang N (2014). Effects of storage and processing on residue levels of chlorpyrifos in soybeans. Food Chemistry.

